# Sustainability inspired fabrication of next generation neurostimulation and cardiac rhythm management electrodes via reactive hierarchical surface restructuring

**DOI:** 10.1038/s41378-024-00754-w

**Published:** 2024-09-09

**Authors:** Shahram Amini, Hongbin Choi, Wesley Seche, Alexander Blagojevic, Nicholas May, Benjamin M. Lefler, Skyler L. Davis, Sahar Elyahoodayan, Pouya Tavousi, Steven J. May, Gregory A. Caputo, Terry C. Lowe, Jeffrey Hettinger, Sina Shahbazmohamadi

**Affiliations:** 1 Research and Development, Pulse Technologies Inc., Quakertown, PA USA; 2 Biomedical Engineering Department, University of Connecticut, Storrs, CT USA; 3 Department of Materials Science and Engineering, Drexel University, Philadelphia, PA USA; 4 Department of Metallurgical and Materials Engineering, Colorado School of Mines, Golden, CO USA; 5 Department of Biomedical Engineering, University of Southern California, Los Angeles, CA USA; 6 Department of Chemistry and Biochemistry, Rowan University, Glassboro, NJ USA; 7 Department of Physics and Astronomy, Rowan University, Glassboro, NJ USA

**Keywords:** Nanoscale materials, Nanostructures

## Abstract

Over the last two decades, platinum group metals (PGMs) and their alloys have dominated as the materials of choice for electrodes in long-term implantable neurostimulation and cardiac rhythm management devices due to their superior conductivity, mechanical and chemical stability, biocompatibility, corrosion resistance, radiopacity, and electrochemical performance. Despite these benefits, PGM manufacturing processes are extremely costly, complex, and challenging with potential health hazards. Additionally, the volatility in PGM prices and their high supply risk, combined with their scarce concentration of approximately 0.01 ppm in the earth’s upper crust and limited mining geographical areas, underscores their classification as critical raw materials, thus, their effective recovery or substitution worldwide is of paramount importance. Since postmortem recovery from deceased patients and/or refining of PGMs that are used in the manufacturing of the electrodes and microelectrode arrays is extremely rare, challenging, and highly costly, therefore, substitution of PGM-based electrodes with other biocompatible materials that can yield electrochemical performance values equal or greater than PGMs is the only viable and sustainable solution to reduce and ultimately substitute the use of PGMs in long-term implantable neurostimulation and cardiac rhythm management devices. In this article, we demonstrate for the first time how the novel technique of “reactive hierarchical surface restructuring” can be utilized on titanium—that is widely used in many non-stimulation medical device and implant applications—to manufacture biocompatible, low-cost, sustainable, and high-performing neurostimulation and cardiac rhythm management electrodes. We have shown how the surface of titanium electrodes with extremely poor electrochemical performance undergoes compositional and topographical transformations that result in electrodes with outstanding electrochemical performance.

## Introduction

Given the growing and aging global population coupled with numerous cardiac^1,2^, neurological^3–6^, retinal^7,8^ and hearing disorders^9,10^, there is a rising need for advanced biomedical devices, healthcare products, and services^11^. Over recent decades, implantable biomedical devices have become essential for many patients worldwide, playing crucial roles in life-critical and life-sustaining functions^12–14^. Notably, long-term implantable neurostimulation devices have become critically important. They help in the diagnosis, monitoring, and treatment of various cardiac, neurological, retinal, and hearing disorders by stimulating nerves, as well as detecting and recording electrical activities from neural tissue. Over the past six decades, these devices and systems have evolved dramatically, proving to be vital tools in the treatment of such conditions^15^. Neurostimulation devices operate by artificially stimulating living tissues with an external electrical signal from a neurostimulator or an implantable pulse generator (IPG) via an implantable metal electrode or microelectrode array, and then across the membranes of specific neural cells or tissues^16,17^. Electrodes used in these applications need to be biocompatible, stable in a biological setting, and electrically conductive, while ensuring they meet the required electrochemical performance within such environments. Effective electrodes or electrode contacts in microelectrode arrays should demonstrate low impedance for sensing and stimulation, high charge injection capacity for safe and reversible stimulation, and increased capacitance for cardiac pacing applications^18–20^. The effectiveness of these electrodes largely depends on two main aspects: (1) electrode material composition and applications, and (2) surface, geometrical, and topographical properties of electrodes.

### Electrode material composition and applications

Over the last few decades, several metals and alloys have been investigated for use as electrodes in neurostimulation devices. These include platinum (Pt), and other platinum group metals (PGMs) such as iridium (Ir) and their alloys *e.g*. Pt10Ir^11^, stainless steel^4,21^, titanium (Ti)^22^, Ni-based superalloys (e.g., MP35N)^4,21,23,24^, tantalum (Ta)^25^, tungsten (W)^26,27^, and gold (Au)^28^. Among these materials, PGMs are the most common electrode materials due to their excellent conductivity, mechanical and chemical stability, biocompatibility, corrosion resistance, radiopacity, and electrochemical performance^11,29–33^. Since the early 1970s, PGMs—and Pt in particular—have been used in medical devices for a variety of cardiac and neurological disorders and other life-threatening conditions. In 2010 alone, ~175,000 oz of Pt was estimated to have been used in biomedical devices^11^. According to the United Nations Environment Programme (UNEP), the global population will reach over 9 billion by 2050 with nearly 90% of the world’s population located in developing countries. Thus, with the ageing and increasing world population, and increasing access to healthcare and advanced medical treatments in developing countries, devices using PGM-based electrodes will significantly contribute to improving the quality of life of people around the globe^11^. Currently there are not any precise reports or data on the volume of PGMs consumed in neurostimulation and cardiac rhythm management devices, but the number of these devices implanted annually is noteworthy. For example, ~1.2 M cardiac pacemakers alone were implanted globally in 2016, with a projection of ~1.5 M to be implanted globally in 2023^34^. As per the World Health Organization 2020 report^35^, more than 264 million adults globally are suffering from various neurological disorders. As a result, the global implantable neurostimulators market size was valued at $4.6 billion in 2019 and is expected to expand at a compound annual growth rate of 11.7% from 2020 to 2027. Additionally, according to the World Health Organization, there are ~430 million people globally suffering from hearing loss or hearing disorders and by 2050 this figure is expected to rise to over 700 million people. On the other hand, the global cochlear implant market was valued at $1.5 billion in 2021 and expected to grow at a compound annual growth rate of 8.7% between 2022 and 2030, reaching $3.1 billion by 2030^36–38^. Since almost all cardiac, neurostimulation, and cochlear implants require PGMs in the manufacture of their electrodes and microelectrode arrays, it is obvious that PGM consumption in these devices is worthy of attention. Most importantly, PGM content in the earth’s upper crust is approximately 0.01 ppm and PGM ore mining takes place in limited geographical areas. In addition, PGM manufacturing processes are extremely expensive, complex, and challenging with possible health hazards. Hence, PGMs are associated with high supply risk and significant economic importance. Consequently, PGMs are classified as critical raw materials, thus their effective recovery or substitution worldwide is of paramount importance^39^. When PGMs are refined, over 95% recovery can be achieved once PGM-containing scraps reach a state-of-the-art refining facility. Among all sectors consuming PGMs, industrial applications are found to lead the way in terms of recycling rates. More importantly, PGM thrifting and substitution has been rapidly growing in various industries due to tightening legislative restrictions in Europe and North America, and high PGM prices^40^. However, in long-term implantable neurostimulation applications—e.g., cardiac pacemakers, implantable defibrillators and other neurostimulation devices—postmortem recovery (from deceased patients) and refining of PGMs that are used in the manufacturing of the electrodes and microelectrode arrays is extremely rare, challenging, and highly costly. The majority, if not all, of these devices remain with deceased patients if the deceased is not cremated. If a deceased patient is cremated, only the IPG that contains the battery is explanted (due to the risks and dangers of battery explosion in crematoria^41^) at funeral homes and are often donated for reuse in developing countries due to the vast disparities in new device implantation rates between developed nations and developing countries^42–44^. In either case, regardless of whether the deceased will be subjected to cremation or not, the leads, the electrodes, and the microelectrode arrays that contain the PGMs remain in the body and are not explanted for reuse or recovery/refining. Therefore, substitution of PGMs with other materials and/or electrode surface topography that would lead to reduction in the amount of PGM used per patient are the only viable solutions to reduce the use of these metals in long-term implantable neurostimulation applications to ensure sustainability and security of PGM supply chain for the future^45^.

### Surface, geometrical, and topographical properties of electrodes

**Fig. 1:**
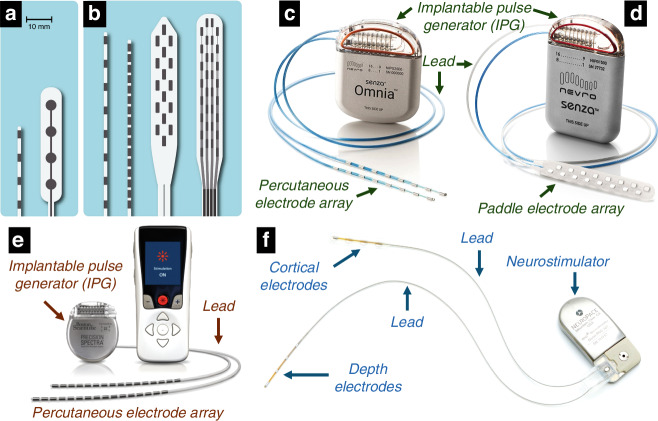
Overview of various types and generations of neurostimulation electrodes and microelectrode arrays. Examples of older generations of percutaneous and paddle electrode arrays that included **a** four electrodes used in spinal cord stimulation devices. **b** Examples of newer generations of percutaneous arrays that include 8–16 electrodes and paddle arrays that include 16–32 electrodes to help improve stimulation selectivity and flexibility in spinal cord stimulation devices [**a**, **b** Photo reproduced with permission from Lempka, S^93^]; **c**–**e** examples of commercial spinal cord stimulation devices for treatment of chronic pain: **c** Omnia™ System, and **d** Senza™ System by Nevro Corp. [**c**, **d** photos used with permission and courtesy of Nevro Corp.]; the Omnia™ System represents a percutaneous style of electrode (Pt10Ir alloy) array with 16 percutaneous (cylindrical) electrodes that is implanted in the spinal cord system. The Senza™ System represents a paddle style of electrode (Pt10Ir alloy) array with 16 flat electrodes that is implanted in the spinal cord system; **e** a spinal cord stimulator system with a percutaneous electrode array that includes 32 cylindrical Pt10Ir electrodes [photo with permission and courtesy of Boston Scientific; ©2022 by Boston Scientific Corporation or its affiliates. All rights reserved.]; **f** an example of a responsive neurostimulation device (RNS® System, NeuroPace, Inc.; photo used with permission and courtesy of NeuroPace, Inc.); this neurostimulator is implanted in the skull, replacing a similarly shaped section of bone. The cortical strip or depth electrodes (Pt10Ir alloy) are implanted in or on the epileptic seizure focus

Typically, electrodes with larger sizes possess a higher geometric surface area (GSA), which enables them to inject more charge before hitting the potential threshold for irreversible electrochemical reactions^18^. Yet, the larger size of these electrodes can diminish their spatial selectivity^46^. To increase the charge injection capacity for delivering a higher resolution electrical signal and/or improving device performance^46,47^, GSA can increase by increasing the number of electrodes. Nonetheless, given the constraints posed by the confined spaces within the brain, spinal cord, cochlea, and eye, adding more electrodes necessitates smaller electrode sizes, which in turn limits the charge each can deliver, negatively affecting device efficacy and countering the purpose of electrode multiplication. A viable solution is to enhance the electrochemical surface area (ESA) of the electrodes, thereby allowing a reduction in GSA while maintaining high charge transfer capabilitiy and low impedance due to the increased electrochemically active surface area^18,46,47^. Optimizing ESA while reducing GSA allows the integration of more electrodes within a device, thus improving performance, selectivity, fidelity, and energy efficiency. Figure 1 shows both old and new generations of electrode arrays in neurostimulation devices, including recent commercial devices with diverse electrode array numbers and geometries.

Over the last 20 years, various methods and technologies have been introduced to enhance the ESA of electrodes, such as iridium oxide thin films (IrO_2_)^18,32,33,48–62^, fractal titanium nitride coatings (TiN)^53,63–65^, black or porous platinum (Pt) coatings^66–69^, conductive polymers^47,70–76^, two-dimensional materials^77,78^, carbon nanotubes^79–82^, and nanostructured scaffolds^83^. Most recently, hierarchical surface restructuring (HSR)^17^ which uses ultrashort laser pulses to create topographical features at multiple length scales on the electrode surface, has demonstrated electrochemical performance values significantly surpassing previous reports. For instance, improvements include a hundredfold increase in charge storage capacity and a more than 700-fold increase in specific capacitance compared to non-restructured Pt10Ir electrodes^17^. Hierarchically restructured Pt10Ir electrodes also show superior in vivo performance, holding considerable promise for neurostimulation applications^84^. Although the HSR method has led to unprecedented performance levels, it still depends on PGMs for electrode fabrication. As previously discussed, replacing PGMs with materials offering comparable or superior electrochemical performance is essential for a sustainable and viable reduction in PGM use in long-term implantable neurostimulation devices.

Among all metallic materials outlined earlier considered for electrode use in neurostimulation devices, titanium (Ti) and its alloys are predominantly utilized in various non-stimulation medical device applications, such as dental and orthopedic implants. This widespread use is attributed to Ti’s biocompatibility, corrosion resistance, and favorable mechanical properties. Despite its electrical conductivity, Ti is not ideal for electrodes and microelectrode arrays due to its tendency to readily form a thin, dense, and non-conductive Ti dioxide (TiO_2_) layer at the electrode-air interface^85^. This layer significantly hinders its electrochemical efficacy, making it a less suitable choice for neurostimulation and cardiac rhythm management devices. Conversely, titanium nitride (TiN) is used extensively as a surface coating for electrodes in cardiac rhythm management devices due to its exceptional electrochemical properties when deposited in a fractal coating form^53,63–65^.

Although coatings can improve the electrochemical performance of electrodes, they present manufacturing and throughput challenges. Coatings are typically not conducive to serial or in-line production processes. Many applications necessitate expensive and lengthy vacuum and batch processes, which are dependent on the required coating thicknesses. These processes can extend over several hours to attain the necessary coating thickness and desired performance. Additionally, coating technologies frequently necessitate the use of masks, which can be expensive and difficult to produce, to selectively apply coatings to specific areas of the electrode surface.

An approach that can be utilized to address some of the shortcomings of coating technologies is laser nitriding, in which short or ultrashort laser pulses are used to synthesize TiN via a reaction between a titanium surface and nitrogen gas from a nitrogen-rich atmosphere. Synthesis and formation of TiN coatings by laser-based approaches in a nitrogen-rich reactive environment have been vastly studied in the literature^86–91^ primarily for wear and corrosion protection in industrial and some medical device applications. The method of direct laser synthesis in a reactive gas environment represents an alternative to the production of hard TiN coatings deposited by classical manufacturing techniques. In the studies reported in the literature^86–91^, a wide range of lasers, e.g., free electron, excimer, nanosecond, and femtosecond lasers, have been used. It should be noted, however, that a major issue with the reported laser nitriding methods is the fast formation of an oxide layer on the surface of the titanium substrate^86^, which significantly hinders the effective formation of TiN.

## Objectives

In this work, we combine the advantages of laser surface restructuring, coating deposition techniques, and laser nitriding via the novel technique of reactive hierarchical surface restructuring (RHSR). Using this approach, the electrode surface undergoes chemical and topographical/geometrical changes that result in superior electrochemical performance. We demonstrate that Ti—similar to Pt10Ir^17^—can be hierarchically restructured by femtosecond lasers in the presence of nitrogen to promote reactive synthesis of electrochemically stable TiN on the surface of the electrodes and that the resulting electrochemical performances can be comparable or superior to hierarchically restructured Pt10Ir electrodes.

This report not only introduces the RHSR technology but also systematically investigates the reactive restructuring phenomena towards establishing a controllable process that can be leveraged for sustainable production of neurostimulation and cardiac rhythm management electrodes. Our founding hypothesis is that through laser restructuring in flowing nitrogen, the electrode will develop a hierarchically restructured surface dominated by stable TiN. In this process, the native oxide layer previously formed due to the exposure of titanium to ambient atmosphere is removed through laser ablation. The surface undergoes a reactive hierarchical restructuring step in which hierarchical structures comprised of varying length scales are formed. During this step, the hierarchical structure is covered with reactively synthesized TiN that limits formation of insulating TiO_2_ and imparts high electrochemical performance to the electrode. We confirm our hypothesis through a systematic study, which involves surface and subsurface characterization of hierarchically restructured Ti electrodes through multiple modalities of microscopy and spectroscopy, and correlation of the measured structural and compositional properties with the electrodes’ performance. Last but not least, the in vitro and in vivo biocompatibility studies performed on reactively restructured Ti electrodes have shown that these electrodes elicit no cytotoxicity, no irritation and no sensitization.

## Materials and methods

### RHSR via femtosecond lasering

Of the several laser processing parameters that enable surface tunability as described in a previous work^17^, this study focuses on effective fluence, which is directly correlated to the amount and distribution of energy to the surface, i.e., the energy delivered by the laser per unit area and the spatial distribution of that energy across a surface, as defined in Eq. 1. Fluence determines the outcome of the reactive restructuring process and is directly related to overall laser machining time, an important factor in manufacturability and commercial viability.

1 $${Effective\; Fluence}\left(\frac{J}{{{cm}}^{2}}\right)=\frac{{Average\; Power\; of\; Laser}\,\left(W\right)}{{Laser\; Spot\; Size}\,\left({{cm}}^{2}\right)}* {Processing\; Time}\,(s)$$

**Fig. 2:**
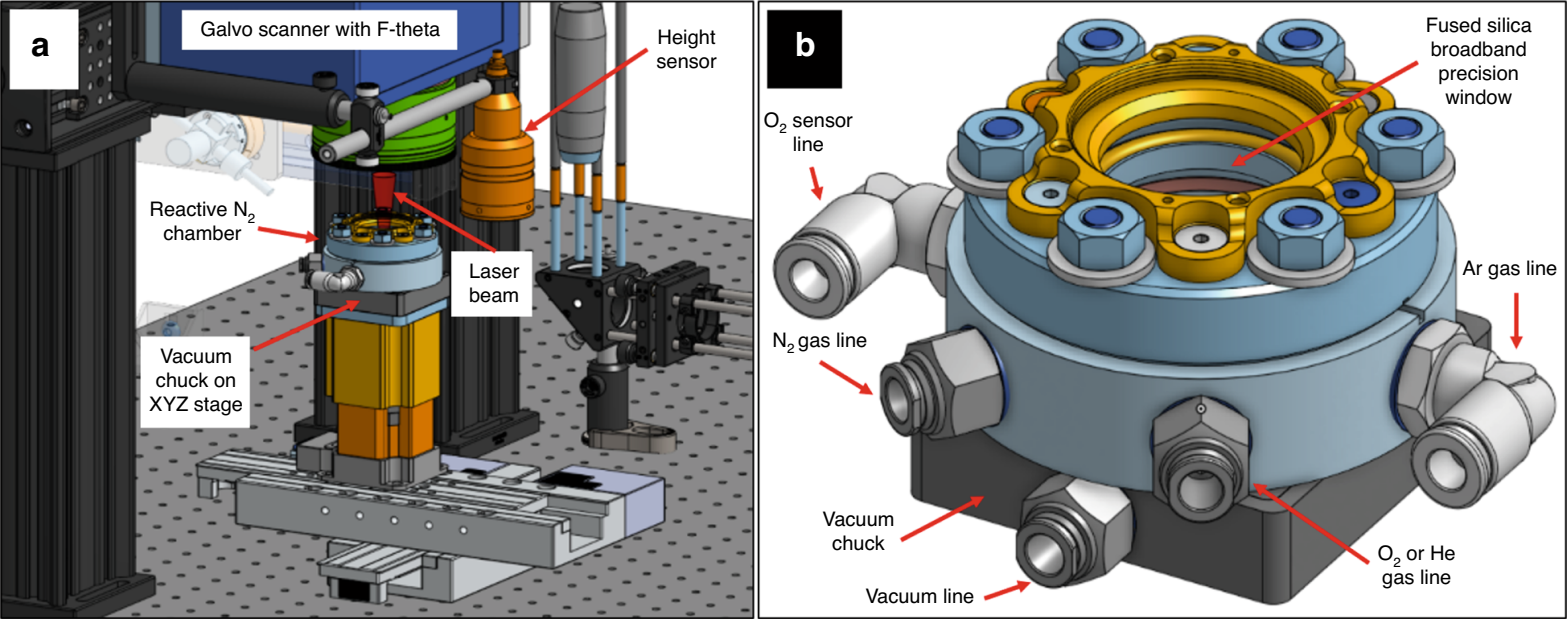
Laser system overview. Overview of **a** laser processing system and **b** reactive N_2_ chamber

The laser source used in these experiments was a Monaco 1035 (Coherent, Santa Clara, CA, USA), that generates 257 fs pulses with a central wavelength of 1035 nm and an initial beam diameter of 2.7 mm. A telecentric, f-theta lens (Wavelength, Singapore) with a focal length of 70 mm focuses the beam down to an ~8 µm spot size at the focal point. The beam is deflected and targeted via an IntelliSCAN galvo scan head (SCANLAB, Pucheim, Germany). In this study, a series of flat 0.5 mm-thick Ti electrodes were hierarchically restructured in a reactive environment (Fig. 2a). The fluences of 89, 51, 39, 33, 29, 24, and 22 MJ/cm^2^ were chosen, while all the other known/controllable lasering parameters were kept constant. The experiments were performed within an oxygen-deprived cylindrical chamber (Fig. 2b) that fed nitrogen gas (N_2_) throughout the RHSR process. N_2_ was supplied through a port in the bottom third of the chamber at a rate of 20 Normal Air Liter per min (NL/min) (gauge calibrated to Ar at 20 °C). The top of the chamber is enclosed with a Ø1” UVFS Broadband Precision Window (Thor Labs, Newton, New Jersey), anti-reflective (AR) coated to accept wavelengths in the range of 350–700 nm. Additionally, as a case study, a Ti leadless pacemaker can was hierarchically restructured circumferentially by mounting the can to a 2-axis rotary (HIWIN GmbH, Offenburg, Germany), positioned so the axis of rotation of the cylinder is set perpendicular to the direction of the laser beam. The focal position of the laser was tuned to top center of the cylinder to minimize deflection. Rotational speed was derived as a function of the can circumference and fluence, calculated so as to permit the laser to traverse the length of the restructured area with theoretically similar overlaps compared to the flat electrodes.

### Confocal and scanning electron microscopy

To qualitatively and quantitatively analyze the surface of reactively restructured Ti electrodes, correlative scanning electron microscopy (SEM) imaging and confocal laser scanning microscopy (CLSM) were conducted. SEM imaging was performed using a ZEISS Crossbeam 340 (ZEISS, Oberkochen, Germany) while CLSM was performed on a Keyence VK 3010 (Keyence, Osaka, Japan). Various surface parameters (root mean square [RMS] height of the surface [*S*_q_], maximum height difference [*S*_z_] and developed interfacial area ratio [*S*_dr_]) as a function of laser fluence were calculated. All SEM and CLSM experimental details and the mathematical equations to obtain these parameters can be found in a previous work^17^.

### X-ray diffraction (XRD) and X-ray photoelectron spectroscopy (XPS) analyses

XRD was performed using a Panalytical Empyrean system (Malvern Panalytical B.V., Almelo, Netherlands) with a copper source emitting *K*_α_ radiation with a wavelength of 1.54 Å in the Bragg-Brentano configuration. A 1/8th degree divergence slit and a 4 mm mask were used during data collection. The incident beam optic was the parallel beam mirror for copper radiation. The detector was a PIXcel3D detector operating in scanning line detector (1D) mode. Data was collected in a range consistent with known peaks from Ti, TiN, and TiO_2_. X-ray photoelectron spectroscopy (XPS) compositional analysis was performed using a PHI VersaProbe 5000 (Physical Electronics, Chanhassen, MN) with Ar^+^ and electron neutralizing flood guns. Ti 2*p* high-resolution spectra were measured between binding energies of 449–474 eV. Depth sputtering was performed using a 1 keV Ar^+^ ion beam rastered over an area of 2 mm by 2 mm. Spectra were analyzed using CasaXPS software with Shirley-type backgrounds applied. The spectra obtained at the surface, prior to sputter etching, were aligned using the adventitious carbon. Following sputtering, this alignment was carried out by shifting the spectra such that the Ti-O 2*p*_3/2_ peak is at a constant energy.

### Focused ion beam (FIB) cross-sectioning and energy dispersive spectroscopy (EDS) analysis

To investigate the depth of restructuring and the morphology of the restructured electrodes, flat restructured electrodes and the anode pacing ring of a Ti leadless pacemaker can were investigated in a Tescan S8252X Plasma FIB system (Brno, Czech Republic) using a Xenon (Xe) plasma. Xe-ions were accelerated with a 30 kV potential difference. Cross-sections were milled and imaged in the dual beam system. Subsequent measurements of composition of the cross sections were performed using EDS in a Thermo Fisher Scientific Apreo S (Brno, Czech Republic) with a tilt stage. The electron source was a thermally assisted field emitter. The Apreo is configured with an Oxford Instruments X-MAX 50 EDS system (High Wycombe, UK) coupled with Aztec v3.3 software. All EDS data were collected using 10 keV electrons and a working distance of 10 mm. After tilting—so that there was a line-of-sight path between the milled region of interest and the X-ray detector—compositional maps were collected. Selected area scans and line scans (not shown) were also collected to confirm compositional gradients which were observed in the maps.

### Ion milling and electron backscatter diffraction (EBSD) analysis

Microstructure, grain size, grain orientation and texture of the restructured Ti electrodes post-restructuring were analyzed with EBSD on a JEOL JSM-7000F Field Emission Scanning Electron Microscope (Tokyo, Japan) equipped with an EDAX Hikari Pro 600pps Detector (Mahwah, NJ). To achieve the surface quality required for EBSD analysis, ion milling was performed on a JEOL IB-09010CP Cross Section Polisher (Tokyo, Japan). The settings used for the ion milling was 5.0 kV accelerating voltage with a 4.5 Argon gas flow resulting in approximately 120 μA current for a duration of 14 h. The EBSD results were analyzed with Ametek Inc.’s OIM Analysis™ version 8 software. Neighbor pattern average re-indexing was performed on the dataset. Grain dilation and neighbor confidence index correlation were performed as the dataset cleanup methods.

### Electrochemical measurements

Cyclic voltammetry (CV) and electrochemical impedance spectroscopy (EIS) were used to measure charge storage capacity (CSC), impedance, and specific capacitance (SC)^17,18,32,33,62^ on reactively restructured Ti electrodes. All CV and EIS experimental details can be found in a previous work^17^.

### Biocompatibility studies

In vitro cytotoxicity against cells in culture was performed in accordance with ISO 10993-5:2009(E). In short, extracts were created by soaking the electrodes in Complete Minimum Essential Medium (Eagles) with Earle’s Balanced Salts, supplemented with 10% fetal bovine serum. These extracts, or controls using USP High-Density Polyethylene Reference Standard or latex beads, were then applied to confluent layers of L929 murine fibroblast cell lines at a ratio of 2 ml extract per 35 mm culture dish. Cells were allowed to grow at 37 °C for 48 h before microscopic evaluation for damaged or rounding cells. The full scoring matrix and methodology can be found in the supplementary information.

In vivo intradermal irritation studies were conducted in accordance with ISO 10993-10:2010(E). In short, healthy female, young adult albino rabbits (NZW/SPF) were injected with polar or non-polar extracts from the electrodes using either saline or vegetable oil as the solvent, respectively. After clipping the fur on the subjects, a series of 0.2 ml injections were made, with 5 polar extract injections and 5 non-polar extract injections on one side of the spinal column. Controls, saline as a negative control and 0.1% SDS in 0.9% sodium chloride as a positive control, were injected on the opposite side of the spinal column. Examination of injection sites was performed immediately upon injection and at 24 h intervals through 72 h post injection. Erythema and edema at the injection sites were assessed using the scoring found in the Supplementary Information, along with a full description of the methodology.

Similarly, in vivo skin irritation tests were carried out in accordance with ISO 10993-10:2010(E). The Guinea Pig Maximization Test (GPMT) was used to assess dermal sensitization using healthy, young adult, albino guinea pigs from a single outbred strain (Dunkin Hartley), weighing between 300 and 500 g. Extracts for the assay were prepared as above. During the induction phase, each subject received 3 injections on either side of the midline with controls being propylene glycol or propylene glycol supplemented with 0.1% dinitrochlorobenzene. Seven days later, the topical induction phase began by placing gel blot paper soaked in the extract or controls over the corresponding injection sites and covered with a bandage. These remained in place for 15 days, at which point the challenge phase began by applying new patches with extracts or controls to each injection site for 48 h. Evaluation for adverse reactions were carried out 48 h after bandage removal using the scoring matrix found in the Supplementary Information, along with a thorough description of the methodology.

## Results and discussion

### Surface and sub-surface characterization

**Fig. 3:**
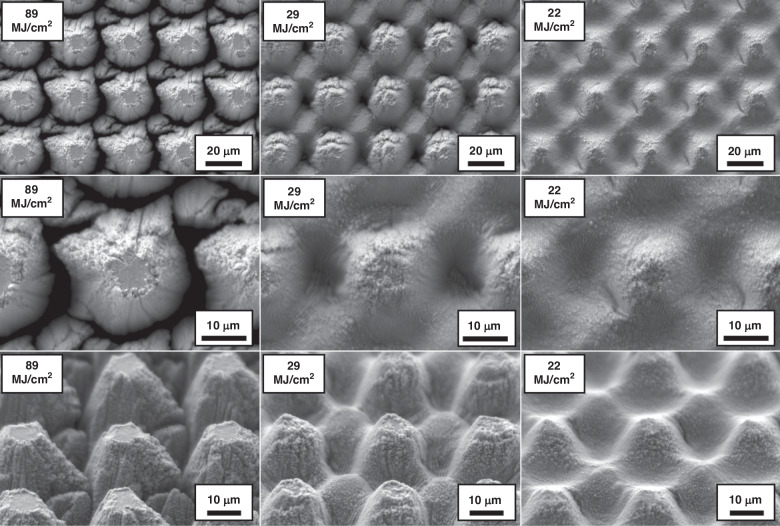
Microstructural results. Representative SEM micrographs of electrodes restructured at 89, 29, and 22 MJ/cm^2^ at two different magnifications (first two rows) and at a 45° tilt angle (third row)

**Fig. 4:**
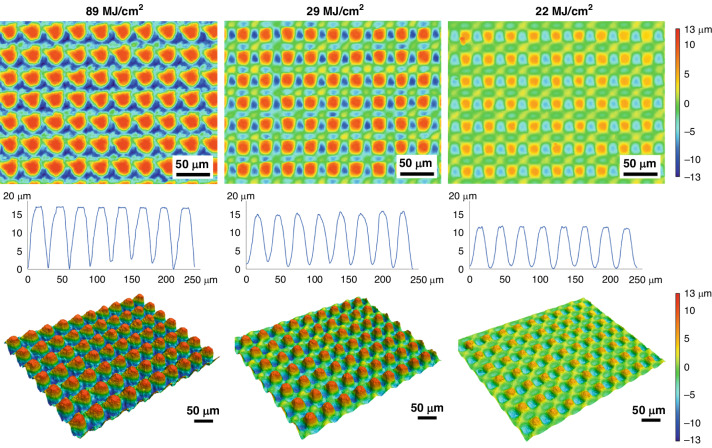
Topographical results. Representative 2D heat maps (top row), cross-sectional profiles (middle row), and 3D reconstructed view (bottom row) of electrodes restructured at 89, 29, and 22 MJ/cm^2^ fluence

**Fig. 5:**
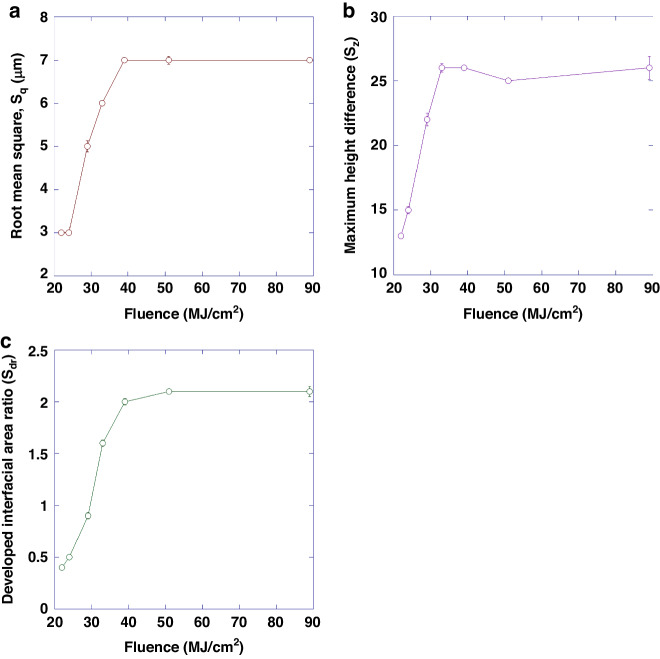
Surface analysis results. Plots of **a** root mean square (*S*_q_), **b** maximum height difference (*S*_z_), and **c** developed interfacial area ratio (*S*_dr_) as a function of fluence

Figure 3 shows representative SEM micrographs of the electrodes restructured at 89, 29 and 22 MJ/cm^2^ fluence at two different magnifications (first two rows) and at a 45° tilt angle (third row). These micrographs qualitatively show that there are larger height differences and higher prevalence of nanoscale features at higher fluences. The first row of micrographs shows the repeatability of the RHSR technique over about 15 peaks, while the second row, with a higher resolution, demonstrates the topographical aspects of a single peak. The third row of micrographs, captured at a 45° tilt angle, displays the shape of each peak and constancy of height. Figure 4 shows representative 2D heat maps (top row), cross-sectional profiles (middle row), and 3D reconstructed view (bottom row) of representative electrodes restructured at 89, 29 and 22 MJ/cm^2^ fluence. These micrographs and profiles quantitatively demonstrate that depth of restructuring is higher at higher fluences, with *S*_z_ being approximately twice as large for 89 MJ/cm^2^ fluence compared to 22 MJ/cm^2^ fluence. Plots of root mean square height (*S*_q_), maximum height (*S*_z_), and developed interfacial area ratio (*S*_dr_) as a function of fluence are shown in Fig. 5. These plots show that *S*_q_, *S*_z_, and *S*_dr_ exhibit a decreasing trend roughly in the 20–40 MJ/cm^2^ fluence regime.

**Fig. 6:**
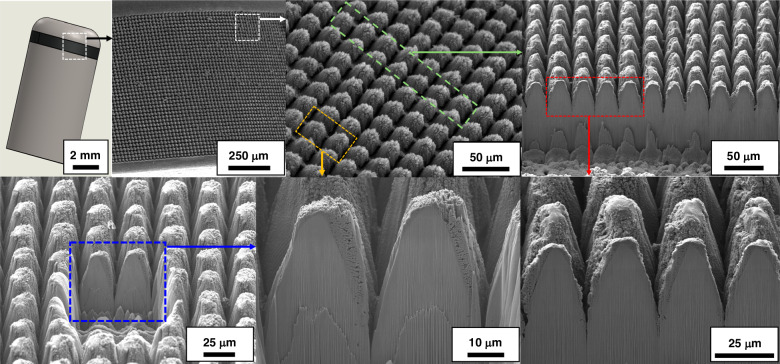
Leadless pacemaker case study. SEM micrographs and FIB cross-sections of the reactively restructured anode pacing ring of the leadless pacemaker exhibiting periodic topography of the reactive hierarchical surface restructuring process

**Fig. 7:**
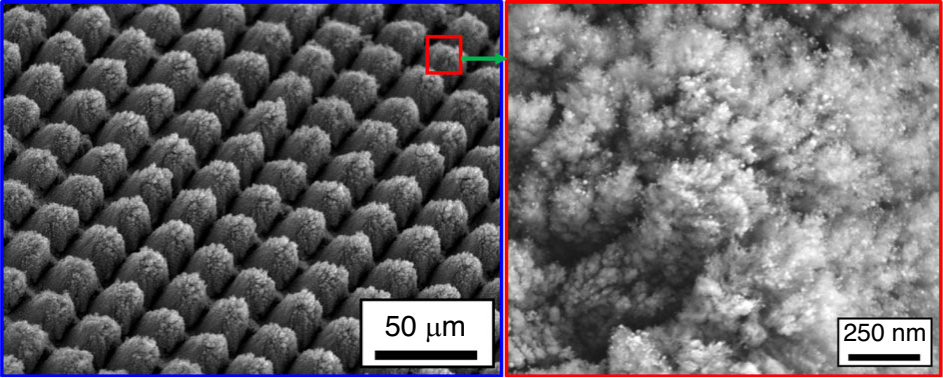
Case study’s microstructural results. SEM micrographs of the reactively restructured anode pacing ring of the leadless pacemaker notably revealing the surface hierarchy and the periodic topography comprised of coarse-scale mound-like features and a finer nano-structure subset on top of the mound-like structures

**Fig. 8:**
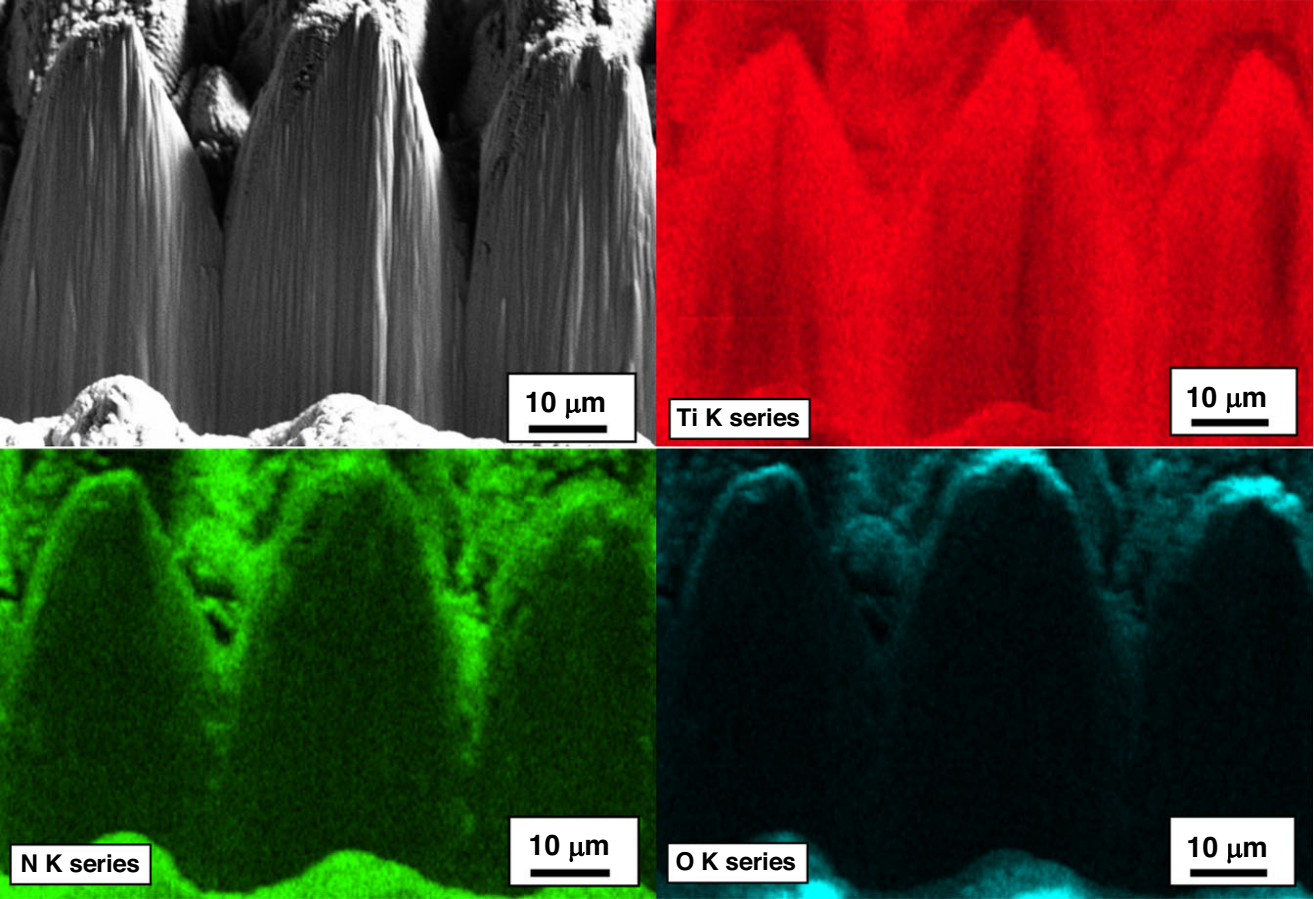
Cross sectional analysis. Milled cross-section of the Ti electrode restructured at 89 MJ/cm^2^ fluence and its associated elemental EDS maps; the nitrogen-rich (green) region is most likely TiN. The oxygen-rich (blue) top surface is likely TiO_2_

Figure 6 presents SEM micrographs and Xe-ion milled cross-sections of the reactively restructured anode pacing ring of the Ti leadless pacemaker can. The hierarchical surface structure created as a result of reactive restructuring can be observed in the SEM micrographs of Fig. 7. The micrographs in Figs. 3, 6, and 7 reveal that the surface hierarchy is notable by a periodic topography comprised of coarse-scale mound-like features that are ~20 µm wide and ~10–30 µm high with a finer nano-structure subset on top of the mound-like structures. In Fig. 6, the lower panel of three micrographs show dense Ti with porous material non-symmetrically formed near the surface of the pillars. Some FIB curtaining effects are noticeable. To understand the origin of the porous materials observed in the milled cross-sectional micrographs, EDS mapping was performed. Figure 8 shows the milled cross-section of the flat Ti electrode restructured at 89 MJ/cm^2^ fluence. In the porous region on top of the pillars, increased nitrogen and oxygen content is noticeable. These nanostructured porous regions are most likely a mixture of TiN and TiO_2_ that are reactively formed and the source of the TiN and TiO_2_ indexed peaks in the XRD and XPS data (discussed below).

**Fig. 9:**
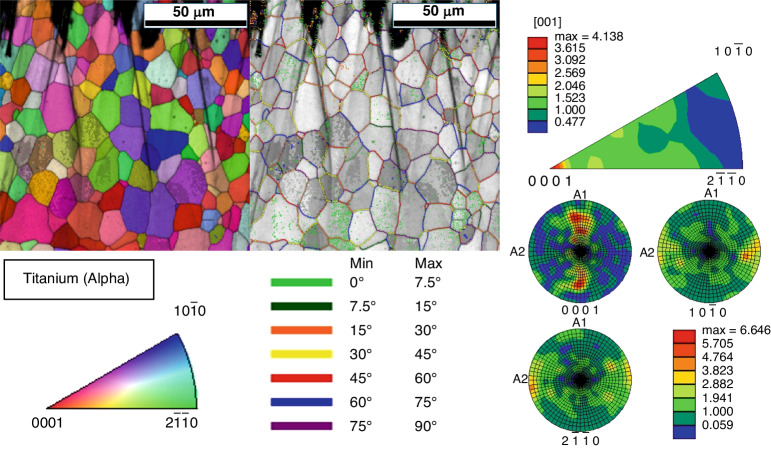
Crystallography. (Left) Inverse pole figure map and IQ/misorientation map of Ti electrode restructured at 89 MJ/cm^2^ fluence; (right) inverse pole figure and pole figure of Ti electrode restructured at 89 MJ/cm^2^ fluence

Analysis via EBSD of the Ti electrode restructured at 89 MJ/cm^2^ fluence revealed a grain size—determined by the average horizontal and vertical intercept lengths—of 5.84 μm. Figure 9 demonstrates the grain structure as shown by the image quality map overlaid with an inverse pole figure map. There is no significant difference in grain size with increasing depth beneath the restructured surface. The grains are equiaxed, with an average aspect ratio of 0.48. Figure 9 also shows the grain boundary misorientations. Most of the grain boundaries have misorientations greater than 15°, with an even distribution between the binned misorientation ranges. There is a moderate diffuse basal texture with respect to the transversely sectioned surface of the substrate, as represented in the inverse pole figure displayed in Fig. 9.

### X-ray diffraction and XPS analysis

**Fig. 10:**
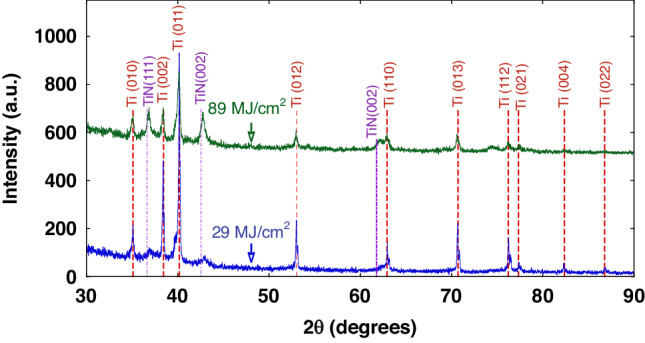
XRD results. X-ray diffraction patterns of Ti electrodes restructured at 89 MJ/cm^2^ fluence (green/top spectra) and 29 MJ/cm^2^ (blue/bottom spectra); dashed lines represent diffraction reference angles for Ti and TiN

Figure 10 shows the results of X-ray diffraction measurements which were made on Ti electrodes restructured at different fluences, showing the overall scan with both Ti and TiN peaks indexed. Only fluences of 89 and 29 MJ/cm^2^ are shown for clarity though all other fluences show a systematic evolution of peak development with the largest TiN (111) peaks observed at 89 MJ/cm^2^ fluence and the smallest peak found at 22 MJ/cm^2^ fluence. It is evident from the diffraction measurements on the electrode restructured at 89 MJ/cm^2^ fluence (Fig. 10) that two TiN peaks emerge at approximately 36.8° (111) and 42.6° (002). These are expected to be the strongest two peaks observed for cubic TiN. It is also evident from Fig. 10 that a peak emerges at approximately 61.8° (022) on the electrode restructured at 89 MJ/cm^2^ fluence. Careful examination of the data reveals that small TiN peaks can be observed in the electrode restructured at 29 MJ/cm^2^ fluence, but the larger peaks observed in the 89 MJ/cm^2^ fluence electrode suggest that more TiN is formed or there is more long-range-order in the electrode restructured at higher fluences. These data confirm that TiN forms when laser restructuring is performed in a nitrogen-rich atmosphere. The differences observed between the electrode restructured at 29 MJ/cm^2^ fluence and those restructured at 89 MJ/cm^2^ fluence are related to the energy delivered per unit area of the electrodes consistent with the overall restructuring observed in Figs. 3 and 4.

**Fig. 11:**
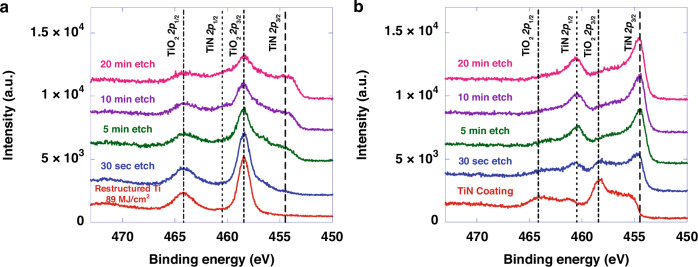
XPS results. X-ray photoemission spectra of **a** Ti electrode restructured at 89 MJ/cm^2^ fluence, and **b** sputter-deposited TiN planar films; spectra obtained after different etch times are offset vertically for clarity to show spectral changes as a function of depth below the surface

Figure 11 shows depth-dependent high-resolution Ti 2*p* X-ray photoelectron spectra (XPS) of a Ti electrode restructured at 89 MJ/cm^2^ fluence (Fig. 11a) and a 4.5 µm-thick sputter-deposited TiN coating on a sapphire wafer (Fig. 11b). The displayed spectral features are deconvoluted to peak pairs attributed to chemically disparate Ti centers of TiO_2_ (~458.9 eV and ~464.7 eV for 2*p*_3/2_ and 2*p*_1/2_ binding energies, respectively) and TiN (~455.0 eV and ~461.0 eV for 2*p*_3/2_ and 2*p*_1/2_, respectively). The surface of the restructured Ti electrode shows a purely TiO_2_ composition, while the data obtained after depth profiling with 1 keV Ar^+^ sputtering contains spectral features associated with both TiN and TiO_2_ compositions. While the non-planar nature of the restructured electrode’s surface complicates quantitative interpretation of the data acquired after depth profiling, the XPS data is consistent with XRD and EDS in confirming a TiO_2_ surface capping the TiN film following restructuring. Meanwhile, the TiN film on sapphire shows a mixture of TiN and TiO_2_ in the surface layer, while depth profiling shows a shift towards pure TiN after 5 minutes of depth profiling. Under these conditions, the sputter rate is expected to be on the order of 1–10 nm/min^92^, indicating that any surface oxide is likely confined to approximately the top 5–20 nm and not more than 50 nm.

### Electrochemical measurements

**Fig. 12:**
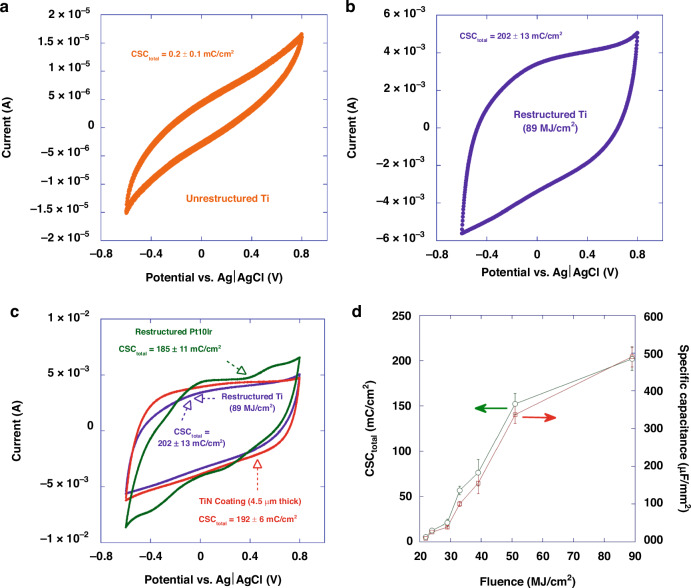
Electrochemical performance results (CV). Representative cyclic voltammograms in room temperature PBS of **a** an un-restructured Ti electrode, **b** a Ti electrode restructured at 89 MJ/cm^2^ fluence, **c** a 4.5 µm-thick TiN coating, restructured Pt10Ir^17^ and Ti electrode restructured at 89 MJ/cm^2^ fluence for the sake of comparison and **d** plot of CSC_total_ and specific capacitance of reactively restructured Ti electrodes as a function of fluence

Figure 12a, b display representative cyclic voltammograms of an as-received un-restructured Ti electrode and a Ti electrode restructured at 89 MJ/cm^2^ fluence, respectively. To provide a better context and for the sake of comparison, Fig. 12c compares cyclic voltammogram of the restructured Ti electrode (89 MJ/cm^2^ fluence) as shown in Fig. 12b with the cyclic voltammograms of a restructured Pt10Ir electrode^17^ as well as a 4.5 µm-thick TiN coating. The un-restructured Ti electrodes exhibited an average CSC_total_ of 0.2 ± 0.1 mC/cm^2^, whereas the reactively restructured Ti electrodes exhibited an average CSC_total_ of 202 ± 13 mC/cm^2^. The restructured Pt10Ir electrodes and the 4.5 µm-thick TiN coating exhibited an average CSC_total_ of 185 ± 11 and 192 ± 6 mC/cm^2^, respectively. The error bars are standard deviation of nine CV measurements, i.e., three electrodes tested three times. Restructured Ti electrodes not only show over three orders of magnitude increase in their CSC_total_ compared to their un-restructured Ti electrode counterparts, but also their CSC_total_ exceeds those of the restructured Pt10Ir electrode^17^ and also a 4.5 µm-thick TiN coating. More notably, CSC_total_ and specific capacitance (SC) seem to be a strong function of fluence for restructured Ti electrodes (Fig. 12d), demonstrating tunability of the electrodes’ performance by adjusting lasering parameters. This is the first time that such performance enhancement and tunability have been reported for restructured Ti electrodes. The restructured Pt10Ir electrodes exhibit an oxidation peak at 0.8 V and a small reduction peak near 0.1 V inherent to Pt10Ir as shown in their cyclic voltammograms that are also semi-rectangular, indicating double-layer capacitance similar to TiN. Restructured Ti electrodes, on the other hand, exhibit a semi-rectangular behavior indicating only double-layer capacitance similar to TiN^17^.

**Fig. 13:**
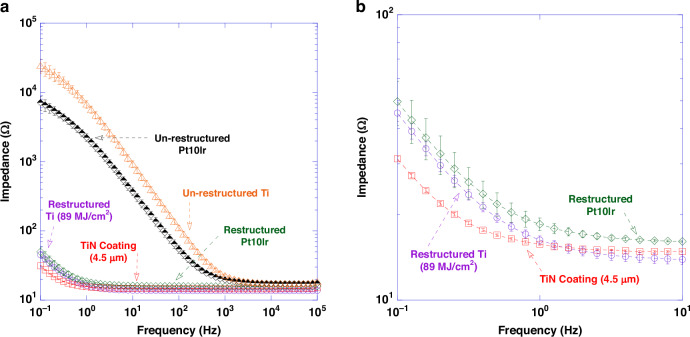
Electrochemical performance results (EIS). **a** Bode plot of impedance magnitude as a function of frequency (plotted in the 0.1–10^5^ Hz frequency range) for un-restructured Ti, restructured Ti at 89 MJ/cm^2^ fluence, un-restructured Pt10Ir, restructured Pt10Ir and 4.5 µm-thick TiN coating in room temperature PBS; **b** Bode plot of impedance magnitude as a function of frequency (plotted in the 0.1–10 Hz frequency range) for restructured Ti at 89 MJ/cm^2^ fluence, restructured Pt10Ir and 4.5 µm-thick TiN coating; Impedance magnitude at 1 Hz demonstrated an average reduction of over 400-fold in the restructured vs. un-restructured Ti electrodes

EIS data measured for un-restructured and restructured Pt10Ir, un-restructured Ti, and restructured Ti (89 MJ/cm^2^ fluence) in room temperature PBS are shown in Fig. 13a (plotted in the 0.1–10^5 ^Hz frequency range). The data is demonstrated in bode-plot format (phase angle not shown) in which the logarithm of the impedance is plotted as a function of the logarithm of frequency. Figure 13b shows Bode plot of impedance magnitude as a function of frequency plotted in the 0.1–10 Hz frequency range for restructured Ti (89 MJ/cm^2^ fluence), restructured Pt10Ir and 4.5 µm-thick TiN coating. Impedance magnitude at 1 Hz and 100 Hz demonstrated an average reduction of over 400-fold and 8-fold in the restructured vs. un-restructured Ti electrodes. At 1 kHz (center frequency of spike activity), the impedance for the restructured Ti (89 MJ/cm^2^ fluence) was reduced by 40% compared to that of the un-restructured Ti electrodes. At high frequencies (greater than 1 kHz and in the 10^4^–10^5 ^Hz frequency range), impedance magnitudes showed resistive behavior representing solution resistance of approximately 14 Ω.

### Biocompatibility studies

The compatibility of the electrodes with host cells and tissues is a critical consideration in the ability to translate these electrodes into clinical and device applications. Several types of biocompatibility measurements was carried out in order to evaluate the electrodes in this context. First, the cytotoxicity against L929 fibroblast cells in culture was evaluated using ISO 10993-5:2009(E). This approach uses extracts made by incubating the electrodes in cell culture media, which are then applied to confluent layers of healthy cells and allowed to incubate for 48 h before microscopic examination. These experiments showed no signs of cellular damage or disruption, with the extracts made from the restructured Ti electrodes performing equally to the negative control (media alone) (Supplemental Fig. SF1).

Subsequently in vivo skin irritation and sensitivity tests were performed using animal models according to ISO 10993-10:2010(E). The skin irritation tests involve intracutaneous injection of polar (saline) or non-polar (vegetable oil) extracts of the electrodes and monitoring the injection sites for erythema and edema. Over the entire 72 h monitoring period, the polar extracts showed no ertythema and edema induction in the subjects, similar to the negative control (saline) (Supplemental Fig. SF2). Non-polar extracts induced no edema responses and only “very slight” erythema over the monitoring period, although the extent of the erythema equivalent to that induced by the negative control (vegetable oil). Together, these results indicate no meaningful skin irritation was caused by the restructured Ti electrodes.

Finally, a skin sensitization assay was performed according to ISO 10993-10:2010(E). This involves initial injections of extracts similar to those made in the irritation study, followed by topical induction by the extracts, followed by the challenge phase and evaluation. The responses are graded using the Magnusson and Kligman Scale for erythema and swelling in response to the challenge phase. In this case, there was no evidence of sensitization caused by either the polar or non-polar extracts (Supplemental Table S4). This is important to ensure long lasting host tolerance of the devices, especially when in contact or penetrating the skin.

## Concluding remarks

In this study, we presented a novel method known as “reactive hierarchical surface restructuring” for manufacturing biocompatible, low-cost, sustainable, and high-performing titanium (Ti) electrodes for next generation neurostimulation and cardiac rhythm management devices and applications. Through multiple modalities of microscopy and spectroscopy for surface and subsurface characterization of hierarchically restructured Ti electrodes, we demonstrated how the surface of Ti electrodes with extremely poor electrochemical performance undergoes compositional, phase and topographical transformations that result in electrodes with superior electrochemical performance. We demonstrated that the surface of the Ti electrodes undergoes reactive hierarchical restructuring in which hierarchical structures comprised of varying length scales are formed while the hierarchical structure is covered with reactively synthesized TiN that limits formation of insulating TiO_2_ that imparts high electrochemical performance to the electrodes. Restructured Ti electrodes not only exhibited over three orders of magnitude increase in their total charge storage capacity compared to their un-restructured Ti electrode counterparts, but also their total charge storage capacity exceeds those of restructured Pt10Ir electrodes. Therefore, the restructured Ti electrodes can inject a larger range of reversible stimulation pulses to the tissue, enabling higher density of electrodes in an electrode array for better selectivity and specificity. Additionally, electrochemical impedance magnitude of the restructured Ti electrodes was also significantly reduced as a result of increasing the effective surface area of the electrodes and the formation of reactively synthesized TiN. This reduction in electrochemical impedance magnitude of the hierarchically restructured Ti electrodes also improves recording performance that renders them highly desirable in neurostimulation and recording applications. Biocompatibility studies were also conducted, demonstrating no cytotoxicity, sensitization, or irritation as a result of exposure to extracts of the restructured electrodes in both in vitro and in vivo model systems. Last but not least, the novel “reactive hierarchical surface restructuring” technology introduced for the first time in this work would lead to reduction and eventual elimination in the amount of PGMs used in long-term implantable neurostimulation and cardiac rhythm management devices, which in turn ensures sustainability and security of PGM supply chain for the future of our planet.

## Supplementary information

Sustainability inspired fabrication of next generation neurostimulation and cardiac rhythm management electrodes via reactive hierarchical surface restructuring

## References

[1] Mulpuru, S. K., Madhavan, M., McLeod, C. J., Cha, Y. M. & Friedman, P. A. Cardiac pacemakers: function, troubleshooting, and management: part 1 of a 2-part series. *J. Am. Coll. Cardiol.* **69**, 189–210 (2017). 28081829 10.1016/j.jacc.2016.10.061

[2] Stevenson, I. & Voskoboinik, A. Cardiac rhythm manegement devices. *Aust. J. Gen. Pract.* **47**, 264–271 (2018). 29779297 10.31128/AJGP-12-17-4439

[3] Epstein, L. J. & Palmieri, M. Managing chronic pain with spinal cord stimulation. *Mt. Sinai J. Med.* **79**, 123–132 (2012). 22238045 10.1002/msj.21289

[4] Ordonez, J., Schuettler, M., Boehler, C., Boretius, T. & Stieglitz, T. Thin films and microelectrode arrays for neuroprosthetics. *MRS Bull.* **37**, 590–598 (2012). 10.1557/mrs.2012.117

[5] Stenehjem, E. & Armstrong, W. S. Central nervous system device infections. *Infect. Dis. Clin. North Am.* **26**, 89–110 (2012). 22284378 10.1016/j.idc.2011.09.006

[6] Schalk, G. & Leuthardt, E. C. Brain-computer interfaces using electrocorticographic signals. *IEEE Rev. Biomed. Eng.* **4**, 140–154 (2011). 22273796 10.1109/RBME.2011.2172408

[7] Kelly, S. K. et al. A hermetic wireless subretinal neurostimulator for vision prostheses. *IEEE Trans. Biomed. Eng.* **58**, 3197–3205 (2011). 21859595 10.1109/TBME.2011.2165713 PMC4876049

[8] Theogarajan, L. Strategies for restoring vision to the blind: current and emerging technologies. *Neurosci. Lett.* **519**, 129–133 (2012). 22414860 10.1016/j.neulet.2012.02.001

[9] Carlson, M. L., Driscoll, C. L., Gifford, R. H. & McMenomey, S. O. Cochlear implantation: current and future device options. *Otolaryngol. Clin. North Am.* **45**, 221–248 (2012). 22115692 10.1016/j.otc.2011.09.002

[10] Wilson, B. S. et al. Better speech recognition with cochlear implants. *Nature* **352**, 236–238 (1991). 1857418 10.1038/352236a0

[11] Cowley, A. & Woodward, B. Healthy future: platinum in medical applications. *Platin. Met. Rev.* **55**, 98–107 (2011). 10.1595/147106711X566816

[12] Stellbrink, C. & Trappe, H.-J. The follow-up of cardiac devices: what to expect for the future? *Eur. Heart J. Suppl.* **9**, I113–I115 (2007). 10.1093/eurheartj/sum071

[13] Halperin, D., Heydt-Benjamin, T. S., Fu, K., Kohno, T. & Maisel, W. H. Security and privacy for implantable medical devices. *IEEE Pervasive Comput.* **7**, 30–39 (2008). 10.1109/MPRV.2008.16

[14] Maisel, W. H. Safety issues involving medical devices: implications of recent implantable cardioverter-defibrillator malfunctions. *JAMA* **294**, 955–958 (2005). 16118386 10.1001/jama.294.8.955

[15] Bazaka, K. & Jacob, M. Implantable devices: issues and challenges. *Electronics* **2**, 1–34 (2012). 10.3390/electronics2010001

[16] Eljamel, S. & Slavin, K. *Neurostimulation : Principles and Practice* (Wiley, 2013).

[17] Amini, S. et al. Femtosecond laser hierarchical surface restructuring for next generation neural interfacing electrodes and microelectrode arrays. *Sci. Rep.* **12**, 13966 (2022). 35978090 10.1038/s41598-022-18161-4 PMC9385846

[18] Cogan, S. F. Neural stimulation and recording electrodes. *Annu. Rev. Biomed. Eng.* **10**, 275–309 (2008). 18429704 10.1146/annurev.bioeng.10.061807.160518

[19] Daubinger, P., Kieninger, J., Unmussig, T. & Urban, G. A. Electrochemical characteristics of nanostructured platinum electrodes-a cyclic voltammetry study. *Phys. Chem. Chem. Phys.* **16**, 8392–8399 (2014). 24664444 10.1039/C4CP00342J

[20] Norlin, A., Pan, J. & Leygraf, C. Investigation of electrochemical behavior of stimulation/sensing materials for pacemaker electrode applications II. Conducting oxide electrodes. *J. Electrochem. Soc.* **152**, J85 (2005). 10.1149/1.1933372

[21] Merrill, D. R., Bikson, M. & Jefferys, J. G. Electrical stimulation of excitable tissue: design of efficacious and safe protocols. *J. Neurosci. Methods* **141**, 171–198 (2005). 15661300 10.1016/j.jneumeth.2004.10.020

[22] Chung, T.-W. et al. Fabrication of iridium oxide/platinum composite film on titanium substrate for high-performance neurostimulation electrodes. *Coatings* **8**, 420 (2018). 10.3390/coatings8120420

[23] Lan, N., Daroux, M. & Mortimer, J. T. Pitting corrosion of high strength alloy stimulation electrodes under dynamic conditions. *J. Electrochem. Soc.* **136**, 947–954 (1989). 10.1149/1.2096892

[24] Schaldach, M. A. X., Hubmann, M. A. X., Weikl, A. & Hardt, R. Sputter-deposited TiN electrode coatings for superior sensing and pacing performance. *Pacing Clin. Electrophysiol.* **13**, 1891–1895 (1990). 1704562 10.1111/j.1540-8159.1990.tb06911.x

[25] Siegfried, J. & Rea, G. *Advances in Neurostimulation Devices* (Springer Berlin Heidelberg, 1988).

[26] Woeppel, K., Yang, Q. & Cui, X. T. Recent advances in neural electrode-tissue interfaces. *Curr. Opin. Biomed. Eng.* **4**, 21–31 (2017). 29423457 10.1016/j.cobme.2017.09.003 PMC5798641

[27] Prasad, A. et al. Comprehensive characterization and failure modes of tungsten microwire arrays in chronic neural implants. *J. Neural Eng.* **9**, 056015 (2012). 23010756 10.1088/1741-2560/9/5/056015

[28] Simmons, W. N., Mackey, S., He, D. S. & Marcus, F. I. Comparison of gold versus platinum electrodes on myocardial lesion size using radiofrequency energy. *Pacing Clin. Electrophysiol.* **19**, 398–402 (1996). 8848386 10.1111/j.1540-8159.1996.tb06509.x

[29] Wellman, S. M. et al. A materials roadmap to functional neural interface design. *Adv. Funct. Mater*. 10.1002/adfm.201701269 (2018). 10.1002/adfm.201701269 PMC5963731 29805350

[30] Normann, R. A. et al. Toward the development of a cortically based visual neuroprosthesis. *J. Neural Eng.* **6**, 035001 (2009). 19458403 10.1088/1741-2560/6/3/035001 PMC2941645

[31] Wang, C. et al. Characteristics of electrode impedance and stimulation efficacy of a chronic cortical implant using novel annulus electrodes in rat motor cortex. *J. Neural Eng.* **10**, 046010 (2013). 23819958 10.1088/1741-2560/10/4/046010

[32] Taylor, G. et al. Electrochemical enhancement of reactively sputtered rhodium, ruthenium, and iridium oxide thin films for neural modulation, sensing, and recording applications. *Electrochim. Acta*10.1016/j.electacta.2021.139118 (2021).

[33] Taylor, G. et al. Investigation of iridium, ruthenium, rhodium, and palladium binary metal oxide solid solution thin films for implantable neural interfacing applications. *Surf. Coatings Technol.*10.1016/j.surfcoat.2021.127803 (2021).

[34] Stewart, C. *Market Report: Global Number of Pacemakers in 2016 and a Forecast for 2023* (Statista Inc., 2022).

[35] World Health Organization. *World Health Statistics 2020: Monitoring Health for the SDGs, Sustainable Development Goals; WHO’s Annual World Health Statistics Report* (World Health Organization, 2020).

[36] Pan American Health Organization. *The Burden of Neurological Conditions in the Region of the Americas, 2000-2019* (Pan American Health Organization (PAHO), 2021).

[37] World Health Organization. *Deafness and Hearing Loss* (World Health Organization, 2023).

[38] Grand View Research. *Cochlear Implant Market Size, Share & Trends Analysis Report* (Grand View Research, 2023).

[39] Yakoumis, I., Panou, M., Moschovi, A. M. & Panias, D. Recovery of platinum group metals from spent automotive catalysts: a review. *Cleaner Eng. Technol.*10.1016/j.clet.2021.100112 (2021).

[40] Hagelüken, B. C. Recycling the platinum group metals: a European perspective. *Platin. Met. Rev.* **56**, 29–35 (2012). 10.1595/147106712X611733

[41] Gale, C. P. & Mulley, G. P. Pacemaker explosions in crematoria: problems and possible solutions. *J. R. Soc. Med.* **95**, 353–355 (2002). 12091510 10.1177/014107680209500708 PMC1279940

[42] Kantharia, B. K. et al. Reuse of explanted permanent pacemakers donated by funeral homes. *Am. J. Cardiol.* **109**, 238–240 (2012). 21996147 10.1016/j.amjcard.2011.08.036

[43] Pavri, B. B. et al. Reuse of explanted, resterilized implantable cardioverter-defibrillators. *Ann. Intern. Med.* **157**, 542–548 (2012). 23070487 10.7326/0003-4819-157-8-201210160-00004

[44] Crawford, T. C. & Eagle, K. A. Reuse of cardiac implantable electronic devices to improve and extend lives: a call to action. *Heart Asia* **9**, 34–35 (2017). 28191825 10.1136/heartasia-2016-010835 PMC5278341

[45] Cowley, A. *PGM Market Report* (Johnson Matthey PLC, 2022).

[46] Green, R. A. et al. Laser patterning of platinum electrodes for safe neurostimulation. *J. Neural Eng.* **11**, 056017 (2014). 25188649 10.1088/1741-2560/11/5/056017

[47] Green, R. A., Lovell, N. H., Wallace, G. G. & Poole-Warren, L. A. Conducting polymers for neural interfaces: challenges in developing an effective long-term implant. *Biomaterials* **29**, 3393–3399 (2008). 10.1016/j.biomaterials.2008.04.047 18501423

[48] Beebe, X. & Rose, T. L. Charge injection limits of activated iridium oxide electrodes with 0.2 ms pulses in bicarbonate buffered saline (neurological stimulation application). *IEEE Trans. Biomed. Eng.* **35**, 494–495 (1988). 3397105 10.1109/10.2122

[49] Harris, A. R., Paolini, A. G. & Wallace, G. G. Effective area and charge density of iridium oxide neural electrodes. *Electrochim. Acta* **230**, 285–292 (2017). 10.1016/j.electacta.2017.02.002

[50] Zeng, Q. et al. Electrodeposited iridium oxide on platinum nanocones for improving neural stimulation microelectrodes. *Electrochim. Acta* **237**, 152–159 (2017). 10.1016/j.electacta.2017.03.213

[51] Negi, S., Bhandari, R., Rieth, L. & Solzbacher, F. In vitro comparison of sputtered iridium oxide and platinum-coated neural implantable microelectrode arrays. *Biomed. Mater.* **5**, 15007 (2010). 20124668 10.1088/1748-6041/5/1/015007

[52] Cogan, S. F., Troyk, P. R., Ehrlich, J. & Plante, T. D. In vitro comparison of the charge-injection limits of activated iridium oxide (AIROF) and platinum-iridium microelectrodes. *IEEE Trans. Biomed. Eng.* **52**, 1612–1614 (2005). 16189975 10.1109/TBME.2005.851503

[53] Weiland, J. D., Anderson, D. J. & Humayun, M. S. In vitro electrical properties for iridium oxide versus titanium nitride stimulating electrodes. *IEEE Trans. Biomed. Eng.* **49**, 1574–1579 (2002). 12549739 10.1109/TBME.2002.805487

[54] Wessling, B. R., Besmehn, A., Mokwa, W. & Schnakenberg, U. Reactively sputtered iridium oxide. *J. Electrochem. Soc.*10.1149/1.2713691 (2007). 10.1109/IEMBS.2007.4353727 18003393

[55] Wessling, B., Mokwa, W. & Schnakenberg, U. RF-sputtering of iridium oxide to be used as stimulation material in functional medical implants. *J. Micromech. Microeng.* **16**, S142–S148 (2006). 10.1088/0960-1317/16/6/S21

[56] Nguyen, C. M. et al. Sol-gel deposition of iridium oxide for biomedical micro-devices. *Sensors* **15**, 4212–4228 (2015). 25686309 10.3390/s150204212 PMC4367406

[57] Slavcheva, E., Vitushinsky, R., Mokwa, W. & Schnakenberg, U. Sputtered iridium oxide films as charge injection material for functional electrostimulation. *J. Electrochem. Soc.*10.1149/1.1747881 (2004).

[58] Cogan, S. F. et al. Sputtered iridium oxide films for neural stimulation electrodes. *J. Biomed. Mater. Res. B Appl. Biomater.* **89**, 353–361 (2009). 18837458 10.1002/jbm.b.31223 PMC7442142

[59] Ullah, N. & Omanovic, S. Large charge-storage-capacity iridium/ruthenium oxide coatings as promising material for neural stimulating electrodes. *Mater. Chem. Phys.* **159**, 119–127 (2015). 10.1016/j.matchemphys.2015.03.061

[60] Cogan, S. F., Plante, T. D. & Ehrlich, J. Sputtered iridium oxide films (SIROFs) for low-impedance neural stimulation and recording electrodes. In *The 26th Annual International Conference of the IEEE Engineering in Medicine and Biology Society*, *San Francisco*, *CA*, *USA*, 4153–4156 10.1109/IEMBS.2004.1404158 (2004). 10.1109/IEMBS.2004.1404158 PMC2709748 17271216

[61] Cogan, S. F., Ludwig, K. A., Welle, C. G. & Takmakov, P. Tissue damage thresholds during therapeutic electrical stimulation. *J. Neural Eng.* **13**, 021001 (2016). 26792176 10.1088/1741-2560/13/2/021001 PMC5386002

[62] Page, N. et al. The effect of deposition parameters on microstructure and electrochemical performance of reactively sputtered iridium oxide coatings. *Mater. Today Commun.*10.1016/j.mtcomm.2021.102967 (2021).

[63] Outten, C. A., Konopka, D. W. & Fennessey, T. F. Development of Titanium Nitride Fractal Coatings for Cardiac and Neural Electrostimulation Electrodes. In *Society of Vacuum Coaters, 57th Annual Technical Conference Proceedings*, *Chicago, IL*, 33–37 (Society of Vacuum Coaters, 2014).

[64] Meijs, S. et al. Electrochemical properties of titanium nitride nerve stimulation electrodes: an in vitro and in vivo study. *Front. Neurosci.* **9**, 268 (2015). 26300717 10.3389/fnins.2015.00268 PMC4523782

[65] Aryan, N. P. et al. In Vitro Study of Titanium Nitride Electrodes for Neural Stimulation. In *2011 Annual International Conference of the IEEE Engineering in Medicine and Biology Society* 2866–2869 (IEEE, 2011). 10.1109/IEMBS.2011.6090791 22254939

[66] Boehler, C., Stieglitz, T. & Asplund, M. Nanostructured platinum grass enables superior impedance reduction for neural microelectrodes. *Biomaterials* **67**, 346–353 (2015). 26232883 10.1016/j.biomaterials.2015.07.036

[67] Li, M., Zhou, Q. & Duan, Y. Y. Nanostructured porous platinum electrodes for the development of low-cost fully implantable cortical electrical stimulator. *Sens. Actuators B: Chem.* **221**, 179–186 (2015). 10.1016/j.snb.2015.06.053

[68] Boretius, T. et al. High-porous Platinum Electrodes for Functional Electrical Stimulation. In *2011 Annual International Conference of the IEEE Engineering in Medicine and Biology Society* 5404–5407 (IEEE, 2011). 10.1109/IEMBS.2011.6091336 22255559

[69] Xia, K., Sun, B., Zeng, Q., Wu, T. & Humayun, M. S. Surface Modification of Neural Stimulating/Recording Microelectrodes with High-Performance Platinum-Pillar Coatings. In *2017 IEEE 12th International Conference on Nano/Micro Engineered and Molecular Systems (NEMS)*. 291–294 (IEEE, 2017).

[70] Green, R. A. et al. Performance of conducting polymer electrodes for stimulating neuroprosthetics. *J. Neural Eng.* **10**, 016009 (2013). 23283391 10.1088/1741-2560/10/1/016009

[71] Green, R. A. et al. Substrate dependent stability of conducting polymer coatings on medical electrodes. *Biomaterials* **33**, 5875–5886 (2012). 22656446 10.1016/j.biomaterials.2012.05.017

[72] Latif, T., McKnight, M., Dickey, M. D. & Bozkurt, A. In vitro electrochemical assessment of electrodes for neurostimulation in roach biobots. *PLoS ONE* **13**, e0203880 (2018). 30303994 10.1371/journal.pone.0203880 PMC6179205

[73] Normann, R. A. & Fernandez, E. Clinical applications of penetrating neural interfaces and Utah Electrode Array technologies. *J. Neural Eng.* **13**, 061003 (2016). 27762237 10.1088/1741-2560/13/6/061003

[74] Du, Z. J., Luo, X., Weaver, C. & Cui, X. T. Poly (3, 4-ethylenedioxythiophene)-ionic liquid coating improves neural recording and stimulation functionality of MEAs. *J. Mater. Chem. C Mater.* **3**, 6515–6524 (2015). 26491540 10.1039/C5TC00145E PMC4610193

[75] Xiao, Y. et al. Electrochemical polymerization of poly(hydroxymethylated-3,4-ethylenedioxythiophene) (PEDOT-MeOH) on multichannel neural probes. *Sens. Actuators B Chem.* **99**, 437–443 (2004). 10.1016/j.snb.2003.12.067

[76] Green, R. & Abidian, M. R. Conducting polymers for neural prosthetic and neural interface applications. *Adv. Mater.* **27**, 7620–7637 (2015). 26414302 10.1002/adma.201501810 PMC4681501

[77] Driscoll, N. et al. Fabrication of Ti_3_C_2_ MXene microelectrode arrays for in vivo neural recording. *J. Vis. Exp.*10.3791/60741 (2020). 10.3791/60741 32116295

[78] Driscoll, N. et al. Two-dimensional Ti_3_C_2_ MXene for high-resolution neural interfaces. *ACS Nano* **12**, 10419–10429 (2018). 30207690 10.1021/acsnano.8b06014 PMC6200593

[79] Wang, K., Fishman, H. A., Dai, H. & Harris, J. S. Neural stimulation with a carbon nanotube microelectrode array. *Nano Lett.* **6**, 2043–2048 (2006). 16968023 10.1021/nl061241t

[80] Voge, C. M. & Stegemann, J. P. Carbon nanotubes in neural interfacing applications. *J. Neural Eng.* **8**, 011001 (2011). 21245526 10.1088/1741-2560/8/1/011001

[81] Ben-Jacob, E. & Hanein, Y. Carbon nanotube micro-electrodes for neuronal interfacing. *J. Mater. Chem.*10.1039/b805878b (2008).

[82] Keefer, E. W., Botterman, B. R., Romero, M. I., Rossi, A. F. & Gross, G. W. Carbon nanotube coating improves neuronal recordings. *Nat. Nanotechnol.* **3**, 434–439 (2008). 18654569 10.1038/nnano.2008.174

[83] Seidlits, S. K., Lee, J. Y. & Schmidt, C. E. Nanostructured scaffolds for neural applications. *Nanomedicine* **3**, 183–199 (2008). 18373425 10.2217/17435889.3.2.183

[84] Li, L. et al. Electrochemical and biological performance of hierarchical platinum-iridium electrodes structured by a femtosecond laser. *Microsyst. Nanoeng.* **8**, 96 (2022). 36065436 10.1038/s41378-022-00433-8 PMC9440118

[85] Bewer, G., Debrodt, H. & Herbst, H. Titanium for electrochemical processes. *JOM* **34**, 37–41 (1982). 10.1007/BF03337977

[86] Hammouti, S. et al. Titanium nitride formation by a dual-stage femtosecond laser process. *Appl. Phys. A* **124**, 411 (2018). 10.1007/s00339-018-1824-x

[87] Fedorov, R. et al. Formation of titanium nitride, titanium carbide, and silicon carbide surfaces by high power femtosecond laser treatment. *ChemPlusChem* **86**, 1231–1242 (2021). 33960734 10.1002/cplu.202100118

[88] Höche, D. & Schaaf, P. Laser nitriding: investigations on the model system TiN. A review. *Heat. Mass Transf.* **47**, 519–540 (2011). 10.1007/s00231-010-0742-z

[89] Schaaf, P., Kahle, M. & Carpene, E. Reactive laser plasma coating formation. *Surf. Coat. Technol.* **200**, 608–611 (2005). 10.1016/j.surfcoat.2005.01.028

[90] Carpene, E., Schaaf, P., Han, M., Lieb, K. P. & Shinn, M. Reactive surface processing by irradiation with excimer laser, Nd:YAG laser, free electron laser and Ti:sapphire laser in nitrogen atmosphere. *Appl. Surf. Sci.* **186**, 195–199 (2002). 10.1016/S0169-4332(01)00625-0

[91] Carpene, E., Shinn, M. & Schaaf, P. Free-electron laser surface processing of titanium in nitrogen atmosphere. *Appl. Surf. Sci.* **247**, 307–312 (2005). 10.1016/j.apsusc.2005.01.059

[92] Greczynski, G. & Hultman, L. Towards reliable X-ray photoelectron spectroscopy: sputter-damage effects in transition metal borides, carbides, nitrides, and oxides. *Appl. Surf. Sci.*10.1016/j.apsusc.2020.148599 (2021).

[93] Lempka, S. F. & Patil, P. G. Innovations in spinal cord stimulation for pain. *Curr. Opin. Biomed. Eng.* **8**, 51–60 (2018). 30911705 10.1016/j.cobme.2018.10.005 PMC6430588

