# Supplementary material for: Sustainability inspired fabrication of next generation neurostimulation and cardiac rhythm management electrodes via reactive hierarchical surface restructuring
Source: Microsyst Nanoeng. 2024 Sep 9;10:125. doi: 10.1038/s41378-024-00754-w (PMC11384795; doi:10.1038/s41378-024-00754-w)
Supplement: Supplementary file 1 — Sustainability inspired fabrication of next generation neurostimulation and cardiac rhythm management electrodes via reactive hierarchical surface restructuring [file 41378_2024_754_MOESM1_ESM.pdf]

Supplementary information

Sustainability inspired fabrication of next generation neurostimulation and cardiac rhythm management electrodes via reactive hierarchical surface restructuring

Shahram Amini *[1,2], Hongbin Choi [2], Wesley Seche [1], Alexander Blagojevic [2], Nicholas May [2], Benjamin M. Lefler [3], *Skyler L. Davis* [4], Sahar Elyahoodayan [5], Pouya Tavousi [2], *Steven J. May* [3], Gregory A. Caputo [6], *Terry C. Lowe* [4], *Jeffrey Hettinger* [7], Sina Shahbazmohamadi [2]

[1] Pulse Technologies Inc., Research and Development, Quakertown, PA 18951
[2] Biomedical Engineering Department, University of Connecticut, Storrs, CT 06269
[3] Department of Materials Science and Engineering, Drexel University, Philadelphia, PA 19104
[4] Department of Metallurgical and Materials Engineering, Colorado School of Mines, Golden, CO 80401
[5] Department of Biomedical Engineering, University of Southern California, Los Angeles, CA 90089
[6] Department of Chemistry and Biochemistry, Rowan University, Glassboro, NJ 08028, USA
[7] Department of Physics and Astronomy, Rowan University, Glassboro, NJ, 08028, USA

***** Correspondence should be addressed to S.A. (email: samini@pulsetechnologies.com)

Supplementary experimental details

Biocompatibility Studies

a) *In vitro* cytotoxicity

Evaluation of cytotoxicity of eluted materials was performed according to ISO 10993-5:2009(E) for cytotoxicity assessment. These tests evaluated the viability of mammalian cells in culture after addition of extracts prepared from restructured Ti electrodes. In this study, L929 murine fibroblast cell line (obtained from ATCC, NCTC clone 929) was chosen as the test system due to its sensitivity and reliability in assessing cytotoxic effects. The L929 cells were grown in culturein Complete Minimum Essential Medium (Eagles) with Earle's Balanced Salts, supplemented with 10% fetal bovine serum and an appropriate antibiotic-antimycotic additives. Confluent cell cultures were trypsinized and re-plated in sterile, tissue culture grade petri plates (35 mm). These plates were grown to create ~80% confluent cell layers by incubating for at least 24 h at 37 ± 1°C in a humidified atmosphere supplemented with 5 ± 1% CO_2. Extracts were created by incubating electrodes (total surface area of 12 cm^2) in the extraction medium at a final ratio of 6 cm^2/ml. Positive controls were created using ratio of 0.2 gm/ml of latex rubber to extraction medium, while the negative control consisted of USP High-Density Polyethylene Reference Standard at an extraction ratio of 3 cm^2/ml. Blanks containing only extraction medium were also prepared. After 24 h of extraction, the extracts were gently agitated and transferred into dry, sterile vessels. No abnormalities or particles were observed, that is, the samples and blanks were clear and particle-free while the latex control extract was cloudy as expected. Therefore, no further processing was needed, and the extracts were immediately used for testing. Subsequently, cultures with approximately 80% confluency and normal cell layer morphology were selected for use in the evaluation. Here, 2 ml of test extract, positive control, negative control, or blank were added to the cell layers in 35mm plates in triplicate. The plates were then returned to incubation for 48 ± 2 h. After incubation, qualitative morphological reactivity was evaluated using the grading system outlined in Table S1. Results are presented in Figure S1.

Table S1: The grading system used to determine qualitative morphological cytotoxicity of the plates following incubation

Grade	Reactivity	Conditions of all cultures
0	None	Discrete intracytoplasmic granules; no cell lysis, no reduction of cell growth.
1	Slight	Not more than 20% of the cells are round, loosely attached, and without intracytoplasmic granules or show changes in morphology; occasional lysed cells are present; only slight growth inhibition observable.
2	Mild	Not more than 50% of the cells are round, devoid of intracytoplasmic granules, no extensive cell lysis; not more than 50% growth inhibition observable.
3	Moderate	Not more than 70% of the cell layers contain rounded cells or are lysed; cell layers not completely destroyed, but more than 50% growth inhibition observable.
4	Severe	Nearly complete or complete destruction of the cell layers

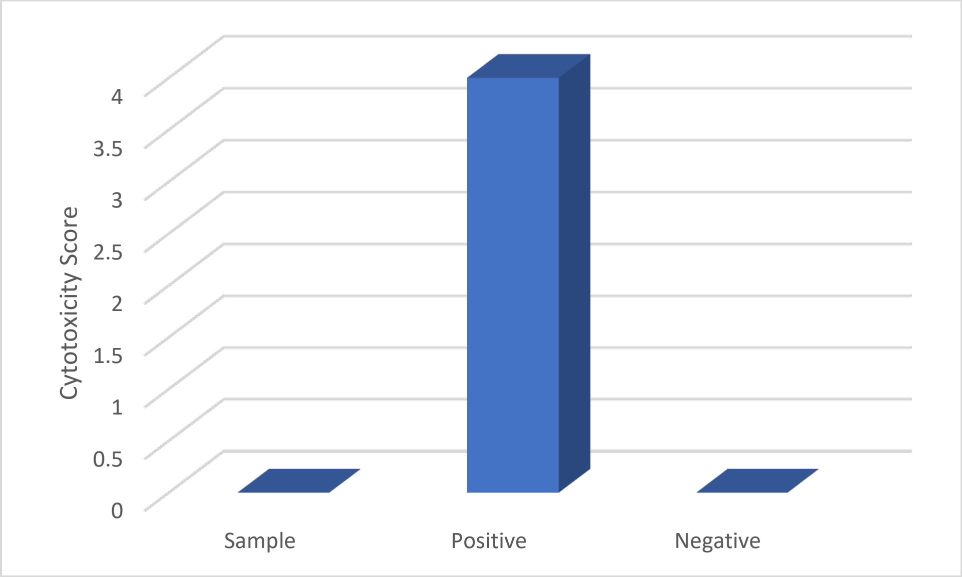

Figure S1: Cell cytotoxicity – Evaluation of cytotoxicity to L929 murine fibroblasts induced by extracts of Ti-HSR electrodes. Extracts or controls were applied to >80% confluent layers of cells, incubated for 48h, and then evaluated microscopically. Cell morphology was evaluated using the metrics in Table SX1. Positive controls consisted of an extract of latex rubber, while the negative control was an extract of USP high density polyethylene reference standard.

b) Intradermal Reactivity

Intracutaneous (intradermal) reactivity assessments were carried out according to ISO 10993-10:2010(E) to assess the potential of the electrodes to induce irritation in response to a single intradermal injection of extracts from restructured Pt10Ir electrodes. Healthy, young adult albino rabbits from a single strain (NZW/SPF), female, weighing not less than 2 kg, previously unused for skin irritation studies, and whose skin was free from mechanical irritation or trauma were used as the test system in this study. Caging and caring of the animals were done in compliance with the Animal Welfare Act, USDA (1995 and subsequent revisions) and the guide for the Care and Use of Laboratory Animals, Institute of Laboratory Animal Resources, National Research

Council, 2011. Electrode extracts were prepared following ISO 10993-12 guidelines. Electrodes with a total surface area of 12 cm^2 were used and extracted using a 6 cm^2/ml ± 10% ratio of electrode to extraction medium. The intact test articles were placed in type I glass extraction vessels, with appropriate volumes of either USP saline (polar extracts) or vegetable oil (non-polar extracts). Controls consisting of only the extraction media were prepared in parallel. Vessels were then warmed to 37 ± 1°C for 72 ± 2 h. After the extraction period, the liquid extracts were agitated to ensure suspension of any particulates and then aseptically transferred into dry, sterile vessels. All sample and control extracts were clear and particle-free upon transfer at this stage. The extracts were stored at ambient temperature and used within 24 hours. Four to eighteen hours prior to the procedure, the fur on each subject's back was closely clipped on either side of the spinal column to expose the skin for testing. The extracts were agitated before being loaded into the syringe and each of three rabbits were given 0.2ml intracutaneously. The injections were delivered such that the polar (saline) test extract was delivered to five sites on the right side of the spine, while the non-polar (vegetable oil) extract was delivered to another five sites on the same side. This pattern was repeated on the opposite side of the subject's spine using the corresponding polar and non-polar blanks. The positive control (0.1% sodium dodecyl sulfate in 0.9% sodium chloride) was intracutaneously injected with 0.2 ml at five sites on the right side of the spinal column, while the negative control (saline diluent) received 0.2 ml intracutaneously at five sites on the left side. Injection sites were evaluated immediately after injection at 24 ± 2, 48 ± 2, and 72 ± 2 h post-injection. Visual inspection evaluated evidence of tissue reaction (erythema and/or edema) at the injection sites. Any reactions were graded on a numerical scale, using the scoring system in Table S2. The individual subject scores for each test sample or blank on an individual subject was calculated by dividing each total by 15 (3 scoring time points x 5 test or blank sample injection sites). Overall mean scores were calculated across the 3 subjects, and the average scores from the blanks were subtracted from the treated mean scores for a final summative score. Results are presented in Table S3.

Table S2: Scoring system for evaluation of skin reactions, as defined in ISO 10993-10:2010(E)

**Erythema and Eschar Formation**	**Score**
No erythema	0
Very slight erythema (barely perceptible)	1
Well defined erythema	2
Moderate erythema	3
Severe erythema (beet-redness) to eschar formation preventing grading of erythema	4
**Edema Formation**	**Score**
No edema	0
Very slight edema (barely perceptible)	1
Well defined edema (edges of area well defined by definite raising)	2
Moderate edema (raised approximately 1 mm)	3
Severe edema (raised more than 1 mm and extending beyond exposure area)	4

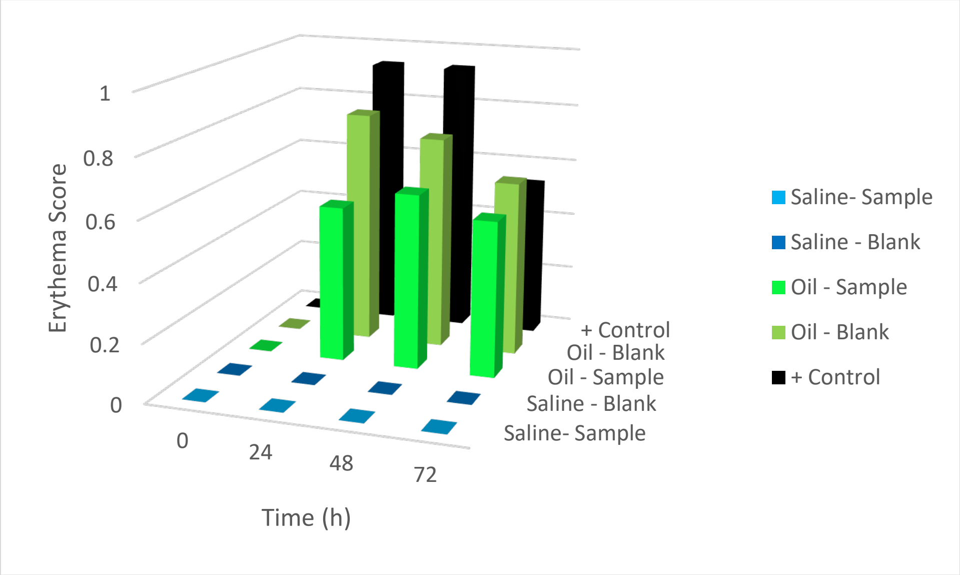

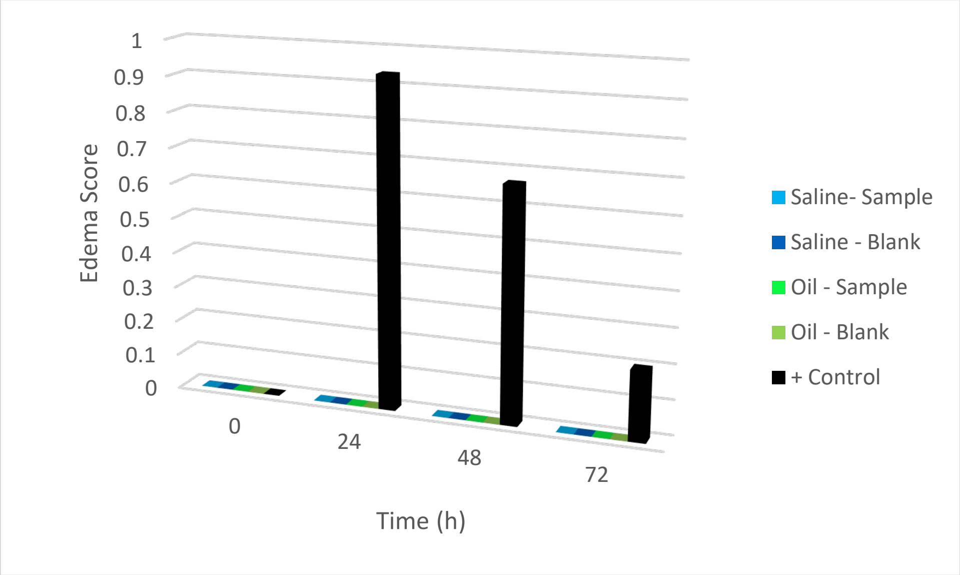

Figure S2 – Intracutaneous reactivity tests assaying (a) erythema or (b) edema at the site of injection. Subjects were injected with extracts made by incubating coated samples in either polar (saline, blue) or nonpolar (vegetable oil, green) medium and subsequently injecting these at 5 different sites on the subject. Erythema and edema was assessed immediately after injection and in subsequent 24-intervals and scored using the metrics in table S2. Positive controls consisted of sodium lauryl sulfate in saline. Blanks are vehicle controls. All data is averages of 3 independent subjects and 5 injection sites per subject.

c) Skin Sensitization

Skin sensitization tests were performed to evaluate the potential of restructured Ti electrodes to elicit contact dermal allergenicity. The Guinea Pig Maximization Test (GPMT) was used to assess sensitization in accordance with ISO 10993-10:2010(E). The study used healthy, young adult, albino guinea pigs from a single outbred strain (Dunkin Hartley) each weighing between 300-500g. The subjects were previously unused for other types of testing. Female animals were nulliparous and not pregnant. The animals were acclimated to the laboratory conditions for a minimum of five days before the start of the experiment. Caging and caring of the animals were in compliance with the Animal Welfare Act, USDA (1995 and subsequent revisions) and the Guide for the Care and Use of Laboratory Animals, National Research Council, 2011. Extracts of the electrodes were prepared as above in the intradermal reactivity tests. The 1:1 mixture of vehicle controls and the test extract was subsequently mixed until emulsified. Before the intradermal induction phase, the intrascapular region of each of the 45 guinea pigs was shaved and cleaned with isopropyl alcohol. Injections were made in a single row of three injections on each side of the midline, within a 4 x 6 cm area. The topical induction phase began 7 days after the intradermal phase. Twenty-four hours before the topical induction phase, the original injection sites were shaved again and with a sample of 10% sodium dodecyl sulfate in petroleum jelly was applied to the areas. The topical induction phase involved a 2 x 4 cm gel blot paper soaked in the electrode test extract (0.5 mL) or controls being placed over the injection sites and covered with an adhesive bandage. These patches remained in place for 48 h. The challenge phase began 15 days after completing the topical induction phase. Immediately before the challenge phase, the flanks of all guinea pigs were clipped free of hair. In all cases, 2 x 2 cm patches soaked in sample extract or controls were used. On the right flank, patches soaked in test extract were applied to the test and negative control groups, while the left flank received a patch with the vehicle alone. Alternatively, for the positive control group patches soaked in 0.1% dinitrochlorobenzene in propylene glycol were applied to the right flank while propylene glycol alone was applied to the left flank. Patches remained on injection sites for 24 $\pm$ 2 h. Visual inspection for adverse reactions at the sites were conducted at 24 and 48 h after bandage removal, following the criteria listed in Table S3.

Table S3: Magnusson and Kligman scale for evaluation of adverse reactions in the skin sensitization studies, as defined in ISO 10993-10:2010(E)

Reaction	Grading Scale
No visible change	0
Discrete or patchy erythema	1
Moderate and confluent erythema	2
Intense erythema and/or swelling	3

Table S4: Skin sensitization tests

Sample	Subject	Sex	24 ± 2 Hour Score		48 ± 2 Hour Score	
			Left	Right	Left	Right
Polar extract	29	F	0	0	0	0
Polar extract	30	F	0	0	0	0
Polar extract	31	F	0	0	0	0
Polar extract	32	F	0	0	0	0
Polar extract	33	F	0	0	0	0
Polar extract	19	M	0	0	0	0
Polar extract	20	M	0	0	0	0
Polar extract	21	M	0	0	0	0
Polar extract	22	M	0	0	0	0
Polar extract	23	M	0	0	0	0
Polar negative control	34	F	0	0	0	0
Polar negative control	35	F	0	0	0	0
Polar negative control	9	M	0	0	0	0
Polar negative control	10	M	0	0	0	0
Polar negative control	11	M	0	0	0	0
Non-polar extract	24	F	0	0	0	0
Non-polar extract	25	F	0	0	0	0
Non-polar extract	26	F	0	0	0	0
Non-polar extract	27	F	0	0	0	0
Non-polar extract	28	F	0	0	0	0
Non-polar extract	14	M	0	0	0	0
Non-polar extract	15	M	0	0	0	0
Non-polar extract	16	M	0	0	0	0
Non-polar extract	17	M	0	0	0	0
Non-polar extract	18	M	0	0	0	0
Non-polar negative control	36	F	0	0	0	0
Non-polar negative control	37	F	0	0	0	0
Non-polar negative control	38	M	0	0	0	0
Non-polar negative control	12	M	0	0	0	0
Non-polar negative control	13	M	0	0	0	0
Positive control (0.1% DNCB)	70	F	0	1	0	2
Positive control (0.1% DNCB)	71	F	0	1	0	2
Positive control (0.1% DNCB)	72	F	0	1	0	2
Positive control (0.1% DNCB)	73	F	0	1	0	1
Positive control (0.1% DNCB)	74	F	0	2	0	2
Positive control (0.1% DNCB)	62	M	0	2	0	2
Positive control (0.1% DNCB)	63	M	0	1	0	1
Positive control (0.1% DNCB)	64	M	0	1	0	2
Positive control (0.1% DNCB)	65	M	0	2	0	2
Positive control (0.1% DNCB)	66	M	0	2	0	2
Positive control vehicle	75	F	0	0	0	0
Positive control vehicle	76	F	0	0	0	0
Positive control vehicle	67	M	0	0	0	0
Positive control vehicle	68	M	0	0	0	0
Positive control vehicle	69	M	0	0	0	0
